# The Microbiome of an Active Meat Curing Brine

**DOI:** 10.3389/fmicb.2018.03346

**Published:** 2019-01-11

**Authors:** David F. Woods, Iwona M. Kozak, Stephanie Flynn, Fergal O’Gara

**Affiliations:** ^1^Biomerit Research Centre, School of Microbiology, University College Cork, Cork, Ireland; ^2^Telethon Kids Institute, Subiaco, WA, Australia; ^3^Human Microbiome Programme, School of Pharmacy and Biomedical Sciences, Curtin Health Innovation Research Institute, Curtin University, Perth, WA, Australia

**Keywords:** brine, microbiome, food, Wiltshire, characterization, next generation sequencing, molecular gastronomy

## Abstract

Traditional food products are important to our culture and heritage, and to the continued success of the food industry. Many of the production processes associated with these products have not been subjected to an in-depth microbial compositional analysis. The traditional process of curing meat, both preserves a natural protein source, as well as increasing its organoleptic qualities. One of the most important salting processes is known as Wiltshire curing. The Wiltshire process involves injecting pork with a curing solution and immersing the meat into microbial-rich brine which promotes the development of the distinct organoleptic characteristics. The important microbial component of Wiltshire brine has not been extensively characterized. We analyzed the key microbial component of Wiltshire brine by performing microbiome analysis using Next Generation Sequencing (NGS) technologies. This analysis identified the genera, *Marinilactibacillus, Carnobacterium, Leuconostoc*, and *Vibrio* as the core microflora present in Wiltshire curing brine. The important food industrial applications of these bacteria were also assessed. The bacterial diversity of the brine was investigated, and the community composition of the brine was demonstrated to change over time. New knowledge on the characterization of key microbiota associated with a productive Wiltshire brine is an important development linked to promoting enhanced quality and safety of meat processing in the food industry.

## Introduction

Traditional food products are of special importance with regards to culture, identity and heritage. Within Europe especially, there is a strong focus on defining the compositional characteristics of traditional products to safeguard these resources for the future (Guerrero et al., 2009). The process of curing food is a traditional method that has been developed over many years, where a mixture of salts and water is used to preserve and flavor foods. This food curing process dates back to ancient times and has been documented by Persian, Greek, and Roman cultures. Wet curing has been applied to a wide range of food, such as vegetables, cheese, fish, and meat products. The curing of meat is important, as meat is a valuable source of protein which decomposes rapidly when not preserved (Pereira and Vicente, 2013; Wojnowski et al., 2017).The defining parameters of the curing process includes the raw product, the environment, the salt concentration, the pH and the temperature. Variation in these parameters define the subsequent microbial communities that develop within the brine. In recent years, there has been a resurgence in wet cured meats, focusing more on a Molecular Gastronomy (MG) approach, where the chemical components (often formed by bacterial actions) are the elicitors of the sensory perception (Barham et al., 2010).

The Wiltshire curing process is one of the foremost methods used for wet curing meat. This traditional process is very well documented and has a rich history with regards to pork products. The Wiltshire process involves injecting whole sides of pork with a curing solution and subsequently immersing the meat in a microbiologically active brine. The meat is immersed for 4–5 days to allow the unique organoleptic properties to develop. Distinct from other curing methods, Wiltshire brine is retained and replenished to maintain an active live bacterial population (Dempster, 1972). Wiltshire curing outlines the necessity of maintaining a salt-tolerant microflora which controls the metabolism of nitrogen within the brine (Andersen, 2004). Bacterial levels have previously been identified at a concentration range of 10^6^–10^7^ CFU/ml (Devine and Dikeman, 2014). Brine used in Wiltshire cured products has a specific microflora which aids in the processing of the meat and this Wiltshire curing method has been given special recognition by the European Union in Regulation 1333/2008 under Annex II in the food category 8.3.4.1. There is limited investigation into brine used to cure meat and large information gaps exist within this traditional process with further comprehensive characterization needed (Eddy and Kitchell, 1961; Gardner, 1971).

Traditional food industries which employ different forms of curing processes are a rich source of unique microflora. However, many food industries that employ live microorganisms experience problems with standardization of their final products (Nediani et al., 2017; Randazzo et al., 2017). Studies recognize the drawbacks of using uncharacterized and unmanaged microbial communities to produce food. To combat this problem, the beneficial microorganisms in the given environment should be monitored closely for any variation. The beneficial microbes can also be isolated and used to create an autochthonous starter culture. The use of starter cultures helps to reduce economic losses and to standardize final products. Commercial starter cultures usually contain one or two strains of bacteria and/or yeast which were identified as the dominant species in the original food environment. Recent research into starter cultures noted that in the future we should focus on the interactions, not only between microbes and the food, but also on the complex interactions within the microbial communities themselves (Leroy et al., 2006). Bacterial communities develop synergistic and antagonistic relationships between each other, with many strains of bacteria depending on the metabolic products of other strains to survive. These interdependencies should be investigated and considered during the formulation of starter cultures, and moreover these interdependencies can be part of the flavor development. The contribution that bacteria make to the development of flavor in cured meat products is recognized by many food industries around the world. Lactic Acid Bacteria (LAB) and Coagulase Negative Cocci (CNC) are especially valued for their ability to improve the flavor, quality, and safety of cured meats (Ammor and Mayo, 2007; Fontana et al., 2016).

Next Generation Sequencing (NGS) has been widely used in recent years, especially in the medical and environmental ecosystems. Microbiome analysis utilizes NGS technologies to sequence fragments of the 16S rRNA marker gene. There has been a huge emphasis on applying this technology to investigate food microbial ecosystems (Bokulich et al., 2016). This technology provides the ability to monitor the relative abundance of organisms present, as well as giving an in-depth investigation into the community dynamics and temporal fluctuations of the microorganisms. Fermented foods have previously been investigated by microbiome analysis with great success, for example in the dairy industry (Walsh et al., 2016; Zalewska et al., 2017), the vegetable industry (Hong et al., 2016) and the meat industry (Fontana et al., 2016). Within the meat industry there has been a number of investigations into dry curing (Greppi et al., 2015; Polka et al., 2015; Ramos et al., 2017), however brine, as that used in the production of Wiltshire cured hams, has had little examination and characterization. Furthermore, studies often focus on detrimental bacteria (den Besten et al., 2017), while our goal is to characterize the beneficial bacteria by microbiome analysis in a productive brine.

It is now emerging that the maintenance and monitoring of an established resident microflora in brine is as important as the detection of detrimental bacteria. Wiltshire brine is a challenging environment promoting strong competition amongst resident microflora. However, as in every environment, bacteria in brine strive to achieve a balance/homeostasis, where certain groups of bacteria occupy specific niches. Maintaining a healthy composition of beneficial bacteria that have been sustained for a number of years, will aid with the control of detrimental or hazardous bacteria establishing themselves in an environment (Man et al., 2017). Meat curing is the least investigated area of food processing using NGS technologies (De Filippis et al., 2017). Our research analyzed Wiltshire compliant brine sampled from an active production facility. A rich microbial community was identified and characterized, and a signature profile of an active Wiltshire brine was defined. The importance of investigating brine has been outlined, which had been neglected for an in-depth analysis, until now. The outcome of this investigation proposes that rigorous, regular analysis should be conducted in order to maintain an optimized brine. While much research focuses on the identification of detrimental bacteria, our study highlights the necessity for maintenance of a healthy beneficial microflora within brine for fully realized production optimization. Disruption in a homeostatic microbiome can lead to the emergence of unwanted and potentially hazardous bacteria, which will also lead to variability in a product and monetary losses.

## Materials and Methods

### Sampling of a Wiltshire Curing Brine for Microbial Content

Wiltshire compliant brine was sampled to investigate the microflora present. Samples were collected from a fully functional curing facility located in the Republic of Ireland. All meats cured in the brine have been consumer assessed and are compliant with normal factory procedures. Curing procedures were compliant with the Wiltshire curing process. Prior to sample collection, brine was stirred with a sterile implement. Brine samples were collected in a blinded study from nine randomly selected 280L curing containers in 2014, as well as three further samples, taken 2 years later in 2016 from the same facility to validate the stable nature of the microbial content of Wiltshire curing. Brine samples were also collected longitudinally during the brining process beginning with brine that was replenished using half old and half fresh brine (Day 0), followed by sampling points at Day 20 and Day 40 after replenishment. These longitudinal samples were collected in 2014. Meat was cured in all brine as normal. All brine was sampled in triplicate to ensure sampling accuracy. Controls were added to ensure experimental accuracy, such as microbiological analysis of the brine ingredients.

### Culture Independent Approach for the Investigation of Brine

We conducted a culture independent approach in order to identify the total community composition within the traditional brining process. Brine (1 ml) was pelleted by centrifugation at 13,000 × *g* for 5 min and the total genomic (g)DNA isolated following manufacturer’s guidelines using the Puregene DNA Extraction Kit (QIAGEN). The gDNA was quantified using a Qubit 2.0 Fluorometer (Life Technologies) and the integrity assessed on a 0.5% agarose gel (Sigma). The gDNA was sent to MWG Eurofins (Ebersberg, Germany) for microbiome analysis. In brief, a tagged 16S rRNA gene PCR for the v3-v5 region was conducted and the region sequenced to 2 × 300 bp on a Next Gen Illumina MiSeq (V3). Bioinformatics were completed by MWG Eurofins, raw reads were demultiplexed based on unique forward and reverse sequences and only high-quality reads were maintained, all reads with sequencing errors were removed. Sequences were assigned to an Operational Taxonomic Unit (OTU). Each OTU represents a distinct cluster, divergent from other sequence clusters. The calculation of alpha diversity including Shannon diversity index, Chao 1 richness and Heip’s evenness index were performed in our laboratory using QIIME 1.9.0 (Caporaso et al., 2010). Microbiome profiles were formed with the percentage relative abundance of the reads relative to the total per sample. The percentage relative abundances of less than 1% individually for less than two of the replicates were categorized together within the “Other” group. For comparisons between the 2 years a global signature profile was constructed using the averages of the OTUs as a percentage of the total. A One-way ANOVA with a *post hoc* Tukey’s Multiple Comparison Test was used to test for significant differences between samples (GraphPad Prism software version 5).

### Culturing the Genera Identified by Microbiome Analysis

A culture dependent approach was combined with molecular diagnostics to examine if genera identified by the microbiome are culturable. To isolate the strains that were present at >1% relative abundance by microbiome analysis, we cultured colonies on Tryptic Soy Agar (TSA) (Merck) ± additional 6% NaCl (Sigma), de Man, Rogosa, Sharpe (MRS) media (Sigma), Marine Agar (Difco) and Luria-Bertani (LB) (Sigma) + 1% NaCl (Sigma) from the same brine samples used for the microbiome study. The Total Viable Count (TVC) was conducted on TSA (Merck) ± 6% NaCl (Sigma). All plates were incubated at 23°C for 2 weeks. Distinct bacterial morphologies were re-cultured to isolate a pure culture. Classification of the distinct morphotypes was based on a number of colony characteristics such as size, surface, texture, color, elevation, and margin (Boone et al., 2005).

Genomic DNA was isolated using the Gentra Puregene DNA Isolation Kit (Qiagen) and the v3-v5 region of the 16S rRNA gene was amplified using a previously published primer set (Marchesi et al., 1998). PCR was completed using the Q5 Hot Start High-Fidelity Polymerase system (New England Biolabs) with the following thermocycler conditions, initial activation at 98°C for 30 s and then subjected to 35 cycles of amplification (denaturation at 98°C for 10 s, annealing at 58°C for 30 s and elongation at 72°C for 30 s) followed by final elongation step at 72°C for 2 min (BIOMETRA T3000). The PCR products were visualized on a 0.8% agarose gel stained with SYBR Safe (ThermoFisher Scientific). The amplicons were purified using the QIAquick PCR Purification Kit (Qiagen). Sanger sequencing was conducted by Eurofins Genomics^1^. All alignments for consensus agreement were constructed using the MUSCLE algorithm (UniproUGENE v.1.21.0 software) (Edgar, 2004). Each nucleotide was supported by at least three independently amplified reads. The genera were identified using *in silico* BLAST (NCBI) searches (Altschul et al., 1990).

## Results

### Identification of the Microbial Signature in Brine by Microbiome Analysis

**FIGURE 1:**
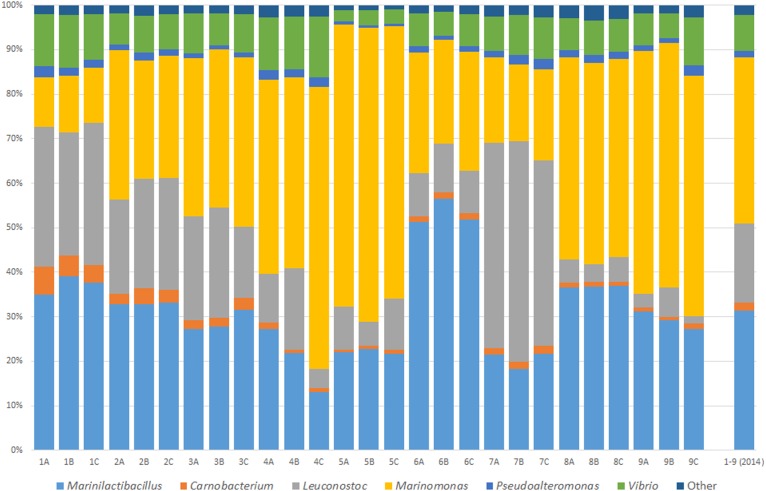
Microbial characterization of an active Wiltshire brine. Brine samples were taken from nine random curing containers in three sampling replicates (A–C) in 2014. Bacterial genera are represented as a relative percentage of the sample’s total microbiome. The microbiome profile of sampling replicates shows minor variations. The composite 2014 microbiome profile illustrates the average bacterial consortium in a Wiltshire brine at this time point. The common and dominant genera in all of the brine sampled, consist of *Marinilactibacillus, Carnobacterium, Leuconostoc, Marinomonas, Pseudoalteromonas, Vibrio*, and “Other.”

Brine was sampled in order to analyze the diversity and abundance of bacteria present. Samples 1–9 were randomly chosen brine samples from a Wiltshire curing industrial facility. These samples were assessed to identify the bacterial composition of the Wiltshire compliant brine. Three sampling technical replicates (A–C) were collected from each of the brine containers. Physicochemical parameters were recorded at the time of sampling and remained relatively constant and within Wiltshire curing standards. Microbiome analysis identified the bacteria present to the genus level. An overview of genus level identification and the percentage relative abundances of the bacteria present is shown in Figure 1. The major genera present were *Vibrio, Pseudoalteromonas, Marinomonas, Leuconostoc, Carnobacterium*, and *Marinilactibacillus*. The “Other” group (relative abundances of less than 1% individually) were one of the smallest groupings. It is clear from Figure 1 that the replicates are quite consistent, with highly similar microbial composition in each. While all of the profile compositions are consistent for the most abundant genera, the relative levels of each of the genera does differ. The largest differences between samples occur with the genera *Marinomonas, Leuconostoc*, and *Marinilactibacillus*.

**FIGURE 2:**
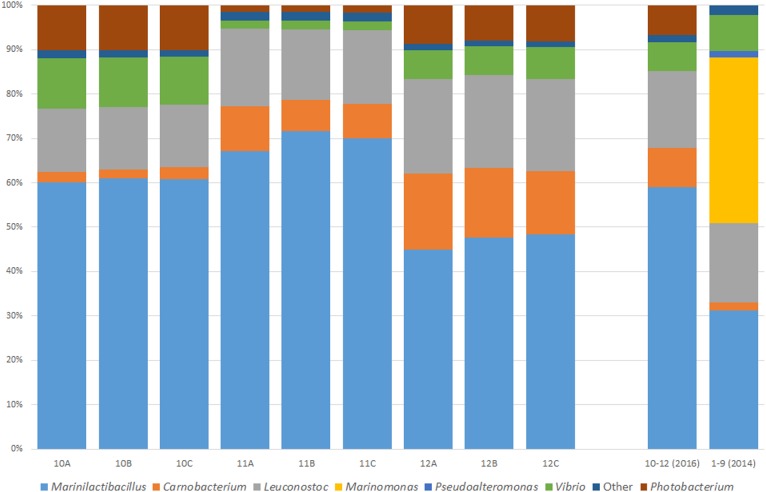
Microbiome of an active Wiltshire brine sampled 2 years after the initial sampling (2016) to test the consistency of the microbiota within the brine. Samples were taken from three random curing containers in three sampling replicates (A–C). Genera are represented as a percentage of the total reads for the sample. Illustrated in this figure is an average bacterial consortium in Wiltshire brine collected in 2014 and 2016. The core microbiome in all of the brine samples over the 2 years consists of *Marinilactibacillus, Carnobacterium, Leuconostoc, Vibrio*, and “Other.” The genera of *Marinomonas* and *Pseudoalteromonas* were not part of the Wiltshire brine core microbiome in 2016, however, *Photobacterium* was present as a dominant bacterium. A microbial signature is present within both the 2014 and 2016 samples, showing a core consistent microbiome of Wiltshire brine.

To assess whether the core microbiome of a Wiltshire brine is consistent and stable, three random brine samples (10–12) were analyzed again in triplicate (A–C) 2 years after the initial sampling point (Figure 2). The main genera present were *Vibrio, Leuconostoc, Carnobacterium, Marinilactibacillus*, and *Photobacterium*. Each of the genera were present as dominant bacteria in the previous sampling point with the exception of *Photobacterium.* This bacterium was in the 2014 samples but at a relative abundance of less than 1%. Most notable was the absence of *Pseudoalteromonas* and *Marinomonas* as dominant bacteria, both of which were now grouped into the “Other” category. A fluctuation did occur with these two genera over the 2-year period, however, *Marinilactibacillus, Carnobacterium, Leuconostoc*, and *Vibrio* consistently appear as the dominant bacteria in both sampling points.

**FIGURE 3:**
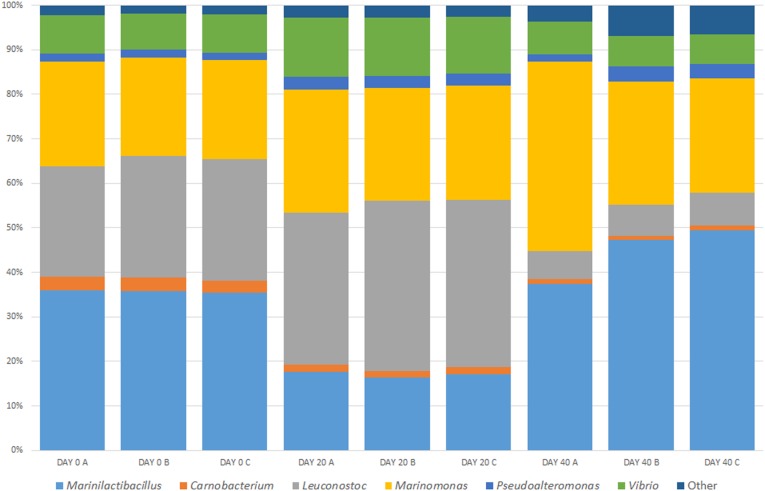
Temporal analysis of an active Wiltshire brine sampled over 40 days. Samples were taken from the same curing container on Day 0, Day 20, and Day 40 in three sampling replicates (A–C). The brine was replenished with half old and half freshly prepared brine (Day 0) and meat cured as normal. As usual practice the “spent” brine is replenished again after approximately 40 days. Before replenishment we sampled the brine for the Day 40 microbiome. The core microbiome in all of the brine samples consists of *Marinilactibacillus, Carnobacterium, Leuconostoc, Marinomonas, Pseudoalteromonas, Vibrio*, and “Other.” While each of the dominant bacteria are present at each timepoint, there are temporal changes observed in each of the genera present.

As well as analyzing nine randomly selected containers of brine, we monitored one container at three time points (in triplicate). The community diversity within this brine was evaluated over three different time points and changes were shown in the microbial community. Figure 3 shows the percentage relative abundance of the bacterial genera present. The dominant bacterial groups previously identified in the brine are again present, namely, *Marinilactibacillus, Carnobacterium, Leuconostoc, Vibrio, Pseudoalteromonas, Marinomonas*, and “Other.” However, there are changes in the microbial diversity in the brine over time. There is a decrease in the percentage relative abundance of *Carnobacterium* and *Leuconostoc* over time. This proportional decrease in these genera is important as they are food associated bacteria, which may contribute an important function to the curing process. *Vibrio* shows an increase in abundance at Day 20 with a subsequent restoration to the Day 0 levels by Day 40. The opposite trend was observed for *Marinilactibacillus* where the percentage relative abundance dropped at Day 20 with and the levels increasing again approaching the last time point. The levels remained static for *Marinomonas* and there was a slight increase observed in the relative abundance over time for *Pseudoalteromonas*. It is important to note that in the Day 40 brine samples, the “Other” group showed an increase in the percentage relative abundance. This new community of bacteria was dominated by *Photobacterium* and *Acinetobacter*. The diversity profile observed in the bacterial community within the “Other” group was maintained until Day 20 however, there is a noticeable fluctuation in the community dynamic at Day 40.

### Investigation of the Biodiversity in the Brine Samples Over Time

**FIGURE 4:**
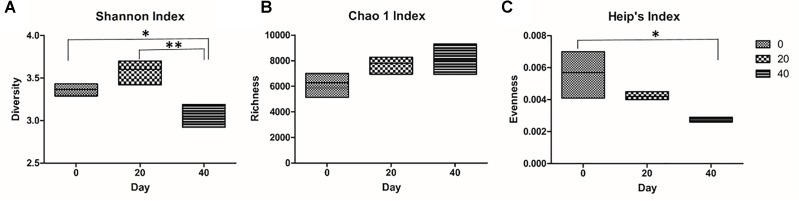
Diversity indexes for the temporal analysis of Wiltshire brine. Three indexes were used to track changes in the microbiome of brine temporally: **(A)** Shannon diversity index, **(B)** Chao 1 richness, and **(C)** Heip’s evenness. The Shannon index of brine sampled on Day 0 and Day 20 differ significantly (^∗^*p* = ≤ 0.05, ^∗∗^*p* = ≤ 0.01, respectively) from brine sampled on Day 40 **(A)**. No statistically significant differences were observed in the Chao 1 richness **(B)**. Heip’s evenness index was significantly different between brine sampled on Day 0 and Day 40 (^∗^*p* = ≤ 0.05) **(C)**.

The microbiome profile of a fully functional brine was investigated for changes in biodiversity over a period of 40 days. The sample coverage was first estimated using Good’s formula. The three time point brine samples exceeded 99% Good’s coverage (Supplementary Table S2). There was no significant difference between the number of OTUs in the Day 0, Day 20, and Day 40 samples (Supplementary Table S2). The diversity present within individual brine samples was measured using the Shannon index. We examined the brine samples for changes in their biodiversity index over time. There was no significant difference observed in the Shannon index between the brine samples collected on Day 0 and Day 20. However, the Shannon index in brine samples from Day 0 and Day 20 differed significantly from that observed from the brine samples at Day 40 (Figure 4A). This difference could be due to the bacterial species classified as “Other” increasing in relative abundance on Day 40. Bacteria classified as “Other” contained genera linked to spoilage and pathogenicity and their propagation in the brine is therefore undesirable. Furthermore, no significant difference in Chao 1 richness was observed between the brine sampled for temporal analysis (Figure 4B). Evenness analysis was preformed using Heip’s index and a statistically significant difference between brine sampled from Day 0 and Day 40 was observed (Figure 4C).

### Culturing the Unique Microbial Signature Bacteria From Wiltshire Brine

Twelve random containers of brine were sampled to analyze the diversity and abundance of bacteria present in Wiltshire brine and a representative microbial signature was consistently present within the brine sampled. Plate counts were conducted to assess the Total Viable Count (TVC) for each of the brine samples (Supplementary Table S1). Sterility and experimental controls were completed for each of the components of brine. No statistically significant levels of bacteria were detected in the ingredient components or the experimental controls. A wide range of TVCs were observed varying from 10^5^ to 10^7^ CFU/ml on TSA and 10^4^ to 10^8^ CFU/ml on TSA supplemented with additional 6% NaCl. There was a high degree of variability in the microbial community in each of the 12 brine samples assessed by the culturable approach employed. As well as the variability in the CFU/ml, there was a consistently higher CFU/ml on the TSA+6% NaCl plates. This may indicate a selection for more halotolerant organisms. With regards the analysis of the brine samples over time, the number of the bacteria present grew from 10^4^ to 10^6^ CFU/ml on TSA and from 10^6^ to 10^7^ CFU/ml on the NaCl supplemented plates.

The recovered colonies on the cultivation plates were screened and profiled for individual colony morphotypes. All individual colony morphotypes profiled and recorded were isolated to a pure culture and a 16S rRNA gene PCR was conducted for taxonomic identification to the genus level. Within the brine, the following genera were present and culturable, *Vibrio, Carnobacterium, Marinilactibacillus, Pseudoalteromonas*, and *Leuconostoc*. The genera *Carnobacterium* and *Marinilactibacillus* were culturable on both TSA ± 6% NaCl and were isolated in all samples at a high abundance. The *Vibrio* genus was cultured on LB+1% NaCl and was present in only a subset of the samples. Strains of *Leuconostoc* were successfully cultured on MRS media. Bacteria of the *Pseudoalteromonas* genera were successfully cultured on Marine Broth. We conducted an extensive literature search for media and conditions to select for and aid in the growth of the *Marinomonas* species and these media and conditions were tested. However, this genus was not culturable by the methods that we applied and may represent an example of a viable but not yet culturable environmental isolate. It is interesting to note that we identified a number of different colony morphologies that were classified as a single genus in our taxonomic analysis. This observation may give an indication that there is more than one species of the genus present in the brine.

## Discussion

### The Value of the Wiltshire Curing Process

The wet curing of meat is very important to the food industry. This process not only adds to the flavor of a meat product but also preserves the much valued protein source (Andersen, 2004). The Wiltshire curing of meat is a historically important process that, until now, has been neglected for detailed investigation into its microbiological component. The microbiological community is an important aspect of the Wiltshire processing of meat (Eddy and Kitchell, 1961; Gardner, 1971). These studies investigated culture dependent microbes associated with the Wiltshire process and their abilities to alter nitrate levels. Both studies also stress the importance of monitoring and maintaining the microbial community. However, these studies have not given a full characterization of the microbes present, as the Wiltshire microbial community was assessed for the TVC and meat-associated flora only. Other important meat curing processes have demonstrated that the microbes present can influence the organoleptic outcome of the final product, for example their contribution to fermented sausage products (Fontana et al., 2016). We have characterized an active Wiltshire curing brine, by applying the powerful tool of NGS to monitor the microbiota in the brine. We have demonstrated that there was a significant difference in the microbiology when the brine was used for 40 days within the curing process without undergoing a replenishment step. The overall core bacteria present consists of *Marinilactibacillus, Carnobacterium, Leuconostoc*, and *Vibrio*. This study also focused on the importance of maintaining beneficial bacteria in a brine community that can outcompete potentially detrimental bacteria introduced by meat and from the environment.

### Application of NGS to the Traditional Curing Food Processes

Traditional food processes have near ubiquitous difficulties with standardization and monitoring of products, and there is a large gap between traditional foods and modern informative technologies (D’Antuono, 2013). We believe that applying the well-studied and powerful technology of NGS to the traditional food industry will aid with production standardization and decreasing monetary losses. NGS has been applied to a number of traditional food processes and successfully identified the key microbial content (Greppi et al., 2015; Polka et al., 2015). NGS gives both an accurate and reproducible account of the microflora present. However, as with any technology, associated limitations such as PCR bias and extraction variation must be acknowledged (Pinto and Raskin, 2012; Weber et al., 2017). Nevertheless, this technology gives an accurate insight into an ecosystem, as well as giving essential data on the relative proportion of each of the bacteria in the communities. This information is crucial for the study of Wiltshire brine as it contains a diverse microbial community and the balance of this community is vital for its function. Traditional culture dependent approaches have been used in previous studies to define the microbial content of a kimchi brine, however, a culture independent sequencing approach appears to give a more accurate profile (Jung et al., 2014).

### The Potential Impact of the Microbiome Signature of Wiltshire Cure

Different microbes possess different biochemical potentials and these contribute specifically to the final sensory properties of the product (Ammor and Mayo, 2007). Hence the maintenance of a specific microbial community with defined microbial proportions is vital for a uniformed final product. The microbiome analysis on the brine from 2 years after the initial sampling showed a great deal of stability over time. The brine was replenished multiple times over the 2-year period with thousands of different hams processed within the wet cures. The microbiome remained stable for the dominant bacterial components. Many of these genera present in the core microbiome have previously been characterized and isolated from other food sources and are of great importance to the food industry. While there were differences between the two microbiomes analyzed over the 2-year period, specifically with *Pseudoalteromonas, Marinomonas*, and *Photobacterium*, these genera are still present in the microbiota, just in different abundances. This may suggest that these do not have a strong contribution to the overall organoleptic qualities. From the data obtained, we suggest that the core microbial signature for a Wiltshire brine consists of *Marinilactibacillus, Carnobacterium, Leuconostoc*, and *Vibrio*. This core microbiota was present in all of the randomly sampled brines sampled over the 2 years. The “Other” grouping of bacteria was one of the most interesting groups, showing a noticeable fluctuation in the community dynamic at Day 40. This should be further investigated to ensure the consistency of the brine at that stage and possibly suggests that the brine be replenished before this point.

Lactic Acid Bacteria often have an important function in food processing through the regulation of pH and flavor development of the final product (Fadda et al., 2010; Randazzo et al., 2017; Martorana et al., 2018). We have identified the LAB, *Leuconostoc* which is capable of producing high quantities of lactic acid (Ju et al., 2016). This bacterial group has also been noted as having the capability to produce diacetyl (Speckman and Collins, 1968). Both lactic acids and diacetyl are very important compounds that contribute to the organoleptic qualities of a wide range of food products. Lactic acid gives a sour/yogurt flavor (Shepard et al., 2013) and diacetyl produces a butter-like flavor in a product (Escamilla-Hurtado et al., 1996). *Carnobacterium* are also LAB and importantly have the capability of growing in temperatures as low as -1.5°C (Jones, 2004). *Carnobacterium* have been isolated from a number of different food sources, including pork, beef, poultry, cheese and seafood (Mauguin and Novel, 1994; Barakat et al., 2000; Cailliez-Grimal et al., 2005; Afzal et al., 2012; Rieder et al., 2012; Youssef et al., 2014). The genus produces a number of secondary metabolites with some of these (specifically class IIa bacteriocins) involved in the inhibition of the detrimental and pathogenic bacteria *Listeria monocytogenes* (Suzuki et al., 2005). With regards flavor, *Carnobacterium* are known to produce compounds that give a malty, buttery and meaty flavor (Miller et al., 1974; Stohr et al., 2001). The *Marinilactibacillus* genera is the third dominant LAB identified in the Wiltshire brine. This genus is usually isolated from the marine environment, specifically in deep seafloor sediment (Toffin et al., 2005). Recently, this genus has been isolated from brine used to cure olives (Lucena-Padros and Ruiz-Barba, 2016). This genera has been identified by culture independent techniques in cheese and assists in the development of this product (Ryssel et al., 2015). Moreover, *Marinilactibacillus* species has antilisterial properties, aiding with the biocontrol of the common food associated pathogen (Roth et al., 2010).

The genera of *Vibrio* are known for their pathogenic strains; however, they also have important functions in the wider environment. *Vibrio* species have been isolated from the natural microflora of Wiltshire curing brine and moreover, the *Vibrio* genus has been previously proven to contribute to the desirable organoleptic qualities of the final product (Petaja et al., 1972; Andersen, 2004). *Vibrio* has been tested directly on pork and improves the taste as well as increasing the levels of aromatic compounds (Hinrichsen and Andersen, 1994). *Vibrio* species are capable of producing 3-methylbutanal which is a compound that contributes a malty/chocolate flavor to a product (Hinrichsen and Andersen, 1994; Afzal et al., 2012; Aprotosoaie et al., 2015).

### Biodiversity and Bacterial Dynamics in the Brine Samples

The biodiversity of the brine samples was analyzed temporally over 40 days. No significant changes in the OTUs and Chao 1 index were observed in the brine sampled at the three time points. Chao 1 is a richness estimator of counts and it provides information on the different species represented in the sample (Chao, 1984). Chao 1 does not take into account the abundances of the species or their relative abundance distributions. We did however see statistical differences in the Shannon index. This parameter provides information about the diversity and is calculated with the number of OTUs and the proportion of the community represented by OTU (Shannon, 1948). The values for Shannon index are usually between 1.5 and 3.5 for ecological data and rarely exceed 4.0 which was true for all of our data sets analyzed (O’Keeffe, 2004). The significant differences in the Shannon index observed between brine samples over time may indicate changes in the proportions of specific bacteria, since there is no statistical difference in OTUs present. We also observed statistical changes in Heip’s evenness. This index is one of many indices developed to measure evenness of live communities, especially variation in rare species (Heip, 2009; Loiseau and Gaertner, 2015). Heip’s index can be defined as a ratio of species diversity to the species richness (Tuomisto, 2012). The statistical analysis of Heip’s evenness index allowed for the detection of significant changes in the relative abundance of rarer genera of bacteria, classified as “Other” in the brine microbiome. These results support our previous observation of changes in the microbiome on Day 40 (Figure 3).

### Culturing the Genera Identified by Microbiome Analysis

The use of NGS increases detection sensitivities and provides a better understanding of microbial communities within complex food environments (De Filippis et al., 2017). In this study, a culture-independent approach gave insight into the relative abundance and the temporal dynamics of bacterial communities within the Wiltshire curing process. Microbiome analysis identified the genera present in the brine, *Marinilactibacillus, Carnobacterium, Leuconostoc, Marinomonas, Pseudoalteromonas, Vibrio, Photobacteria*, and “Other” bacteria. The uncultured data were used to define the optimized culturing conditions for the growth of the bacterial genera. Individual bacterial strains were isolated based on the differences in their colony morphologies, however, this method is based on colony characteristics which can change depending on a large number of variables, such as environmental signals and the culture media (Lebaron et al., 1998). Other limitations of culture-dependent approaches are that they allow only the study of microorganisms that can be cultured in laboratory conditions. Many bacterial strains found in food environments are non-culturable or can enter a “viable but non-culturable” (VBNC) state (Oliver, 2005; Fakruddin et al., 2013). Culture-dependent techniques enabled isolation of all of the bacteria in the 2014 microbiome with the exception of *Marinomonas* which may be a non-culturable strain or be in a VBNC state. The bacterial TVCs of the brine samples range from 10^5^ to 10^7^ CFU/ml on TSA and 10^4^ to 10^8^ CFU/ml on TSA supplemented with additional 6% NaCl. These TVC values are similar to the TVC counts reported for Wiltshire curing brine of 10^6^ to 10^7^ CFU/ml (Andersen, 2004).

Culture-dependent approaches can facilitate the in-depth identification and characterization of the bacterial isolates. It also allows for the investigation into their industrial potential. Culture based techniques offer large quantities of easily sequenced gDNA for the investigation into their metabolic capabilities. Culture techniques also give the opportunity to preserve and store bacterial strains long-term, as a part of a company’s intellectual property (Hansen, 2002; Sisto and Lavermicocca, 2012). Furthermore, cultures can be used to formulate bespoke inoculums which could improve curing or allow targeted flavor manipulation (Fontana et al., 2016; Guarcello et al., 2016; Nediani et al., 2017).

### Solution to Traditional Food Processing Difficulties

The use of a mixed autochthonous starter culture could help with the standardization of the brine by reducing the degree of heterogeneity in the microbial community. While many of the brine samples in this study had a similar profile, there were differences in the profiles of the brines as well as the relative abundances within the brine. Starter cultures have been used extensively in the curing of olives, fish and dairy products so as to ensure a standardized, well monitored product (De Angelis et al., 2015; Guarcello et al., 2016; Speranza et al., 2017). There have also been numerous starter cultures used in the production of meat-based goods. The introduction of starter cultures is becoming the standard method of meat processes due to the positive results and the ease of management (Casquete et al., 2011; Nediani et al., 2017). The use of starter cultures has also improved the safety and the sensory qualities of traditional meat products (Talon et al., 2008). However, these studies into traditional meat products focus on dry cured product, with little or no mention of wet curing. Our study provides a new perspective and data for improving the Wiltshire curing process.

## Conclusion

In conclusion, we report, to our knowledge, the first microbiome study of a Wiltshire brine. This brine has a long tradition in the food industry and gives special recognition to the contribution of the microflora to the curing process. The core microbiota identified in the Wiltshire process consists of *Marinilactibacillus, Carnobacterium, Leuconostoc*, and *Vibrio*. We also noted that there was a difference in the microbial composition of the brine, when not replenished for 40 days. The composition of the microflora in the brine is essential for the quality of the brine and any instability in the community will have an unacceptable effect on the final product (Andersen, 2004). Thus, the characterization and monitoring of the brine is essential and NGS gives a rapid, effective and applicable method to accomplish this goal. We have outlined a microbiota for the development of an inoculum to modernize and standardize the traditional process of Wiltshire curing meat.

## Author Contributions

DW, IK, and FO were involved in the conception, experimentation, and authorship of the work. SF contributed to the experimental processing of the samples as well and contributing to the final manuscript.

## Conflict of Interest Statement

The authors declare that the research was conducted in the absence of any commercial or financial relationships that could be construed as a potential conflict of interest.

## Footnotes

1 www.eurofinsgenomics.eu
